# Teaching Six “blink” features reduces general endoscopist cancer miss rate in image-based assessment of large colorectal polyps

**DOI:** 10.1055/a-2868-4812

**Published:** 2026-05-29

**Authors:** Lynn K. Debels, Tamas Tornai, John T. Anderson, Maria E. Argenziano, Anne Hoorens, Vikash G. Lala, Pieter Jan Poortmans, Sander Smeets, Roland Valori, Lobke Desomer, David J. Tate

**Affiliations:** 1 Department of Gastroenterology and HepatologyUniversity Hospital BrusselsAnderlechtBrusselsBelgium; 2 Medicine and Health Sciences 54498 Ghent University Faculty of Medicine and Health SciencesGhentFlandersBelgium; 3 Department of Gastroenterology and Hepatology 60200 University Hospital GhentGhentFlandersBelgium; 4 Institute of Pancreatic Diseases 37637 Semmelweis University of MedicineBudapestBudapestHungary; 5 Gastroenterology 2379 Gloucestershire Hospitals NHS Foundation TrustCheltenhamEnglandUnited Kingdom of Great Britain and Northern Ireland; 6 9294 Università Politecnica delle MarcheAnconaMarcheItaly; 7 Anatomopathology 60200 University Hospital GhentGhentFlandersBelgium; 8 Department of Gastroenterology & Hepatology 705264 Charlotte Maxeke Johannesburg Academic HospitalJohannesburgGPSouth Africa; 9 Department of Gastroenterology and Hepatology 162942 Wits University Donald Gordon Medical CentreParkstownSouth Africa; 10 Gastroenterology and Hepatology 60201 UZ BrusselBrusselsBelgium; 11 GastroenterologyAZ DeltaRoeselareBelgium; 12 Department of Gastroenterology and Hepatology 60200 University Hospital GhentGentOost-VlaanderenBelgium

**Keywords:** Endoscopy Lower GI Tract, Polyps / adenomas / ..., Diagnosis and imaging (inc chromoendoscopy, NBI, iSCAN, FICE, CLE...), Quality and logistical aspects, Training, Colorectal cancer, Endoscopic resection (polypectomy, ESD, EMRc, ...)

## Abstract

**Background and study aims:**

Accurate cancer detection in large non-pedunculated colorectal polyps (LNPCPs) remains challenging for general endoscopists. We evaluated whether teaching six gross morphological "blink" features could improve accuracy of cancer detection.

**Methods:**

This prospective interventional study assessed general endoscopists evaluating 20 LNPCP images (7 with histologically confirmed submucosal invasive cancer including four deep invasions ≥ 1000 µm, 13 benign). Participants assessed images before and after a 2-minute educational video introducing six blink features: spontaneous bleeding, depression, fold deformation, extra redness, ulceration, and chicken-skin mucosa. Primary outcome was change in miss-rate for cancer detection. Generalized linear mixed models accounted for clustering within raters and polyps.

**Results:**

The 165 participants included gastroenterology consultants (63.6%), trainees (21.2%), students (1.8%) and colorectal surgeons (13.3%) with median colonoscopy experience of 6.5 years. Post-intervention, the cancer miss rate decreased four-fold from 26.6% (95% confidence interval [CI] 13.4–46.0) to 5.7% (95% CI 2.4–12.8). The improvement was consistent across experience levels. The false alarm rate increased less than two-fold from 25.0% (95% CI 15.1–38.5) to 42.2% (95% CI 27.7–58.2). Multivariable analysis identified spontaneous bleeding (odds ratio [OR] 3.92; 95% CI 3.11–4.96), extra redness (OR 3.66; 95% CI 3.09–4.33), and depression (OR 3.06; 95% CI 2.58–3.64) as independent predictors of cancer among general endoscopists. Mean blink features per polyp were 1.08 (95% CI 0.82–1.40) for benign lesions vs 2.46 (95% CI 1.85–3.12) for cancers.

**Conclusions:**

Teaching six blink features to general endoscopists led to a four-times reduction in cancer miss rates in an image-based evaluation. Although specificity decreased, this trade-off favors patient safety because false positives trigger established clinical safeguards whereas missed cancers risk inappropriate endoscopic resection with potentially irreversible consequences.

## Abstract (de)

**Graphical Abstract:**

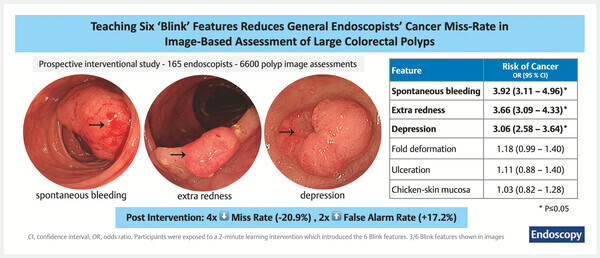

## Introduction

Colorectal cancer is one of the most preventable malignancies through early detection and removal of precancerous lesions via polypectomy 1 2 . The technique required to endoscopically resect a given polyp is related to presence of cancer within that lesion and estimated depth of any submucosal invasion (SMI) 3 . Incorrect decision-making can lead to morbidity and potential mortality for patients and costs for healthcare systems 4 .

Real-time polyp assessment for cancer risk using high-resolution endoscopy with magnification and virtual chromoendoscopy is effective among experts 5 6 , but remains complex for general endoscopists without specific training 7 8 . Because these practitioners will perform the majority of procedures where large (≥ 10 mm) non-pedunculated colorectal polyps (LNPCPs) are first detected, there is a pressing need for simplified and robust approaches to allow estimation of malignant potential and effective triage of treatment strategy.

It is well recognized that human experts, regardless of the domain of expertise, are exceptionally adept at synthesizing large volumes of visual, contextual, and historical information to arrive at a first (or “blink”) impression 9 . In medicine there is precedent for the blink concept. One study 10 demonstrated that expert radiologists who received only brief exposure to mammograms could detect breast cancer as accurately as when they were allowed unlimited exposure to the same mammograms. In another study 11 , both radiologists and cytologists were able to reliably stratify medical images as normal/abnormal during brief exposures (250–2000 milliseconds) without being able to accurately localize the actual pathology during that exposure. Importantly both studies demonstrated that non-experts could not reliably detect the outcome based on brief exposures.

Applied to LNPCPs, success of expert blink impression likely hinges on ability to quickly recognize specific visual features that are strongly associated with underlying malignancy when first encountering the polyp. It follows that these cues would be morphologic features visible from afar and without virtual chromoendoscopy, because there would be no time for detailed analysis of pit/vascular pattern within the required timeframe. So-called gross morphologic features, shown to significantly increase risk of a polyp containing deep submucosally invasive cancer when identified by experts 12 , would be excellent candidates.

**Fig. 1:**
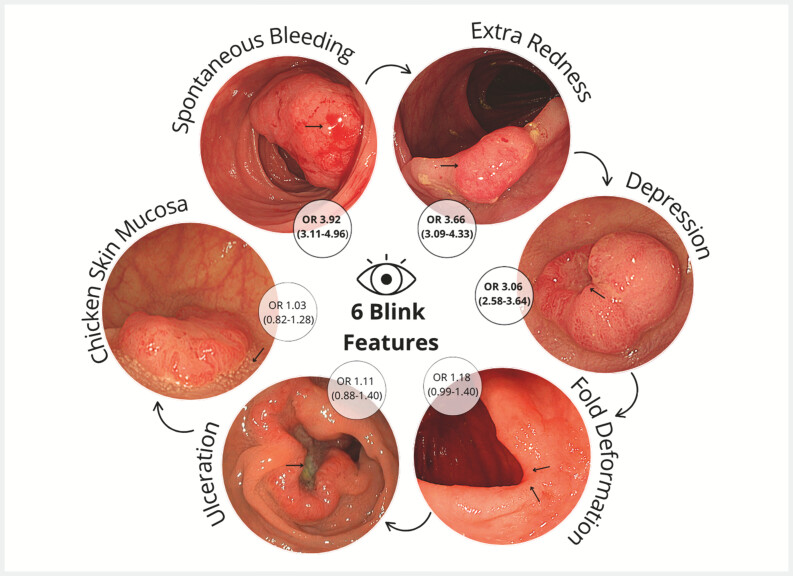
Representative Images of each of the six blink features. White-light endoscopic still images, each captured at ≥ 10 mm distance, demonstrate the six gross morphological “blink” features: spontaneous bleeding; extra redness; depression; fold deformation; ulceration; chicken-skin mucosa. All features are marked by arrowheads. All images were acquired with standard-definition white-light endoscopy and without magnification or virtual chromoendoscopy. Odds ratios (ORs) for cancer detection among general endoscopists with 95% confidence intervals (CI) are shown per blink feature. Statistically significant results ( *P* ≤ 0.05) are indicated in bold.

The authors proposed a condensed set of gross morphological characteristics which could potentially be recognized at a glance by general endoscopists, mimicking the intuitive pattern matching used by experts. It was hypothesized that such an approach could shortcut need for significant experience to identify cancer in LNPCPs. The six selected features, hereafter referred to as “blink features,” were spontaneous bleeding, depression, fold deformation, extra redness, ulceration, and chicken-skin mucosa ( Fig. 1 , **Supplementary Materials-Narrative Review** ) 13 14 15 16 17 18 19 20 21 22 23 24 25 26 27 28 .

This study aimed to assess impact of introducing a structured assessment of the six blink features using a 2-minute learning intervention on diagnostic performance of cancer prediction from still images of LNPCPs among general endoscopists.

## Methods

### Study design

This was a prospective interventional study designed to evaluate accuracy of colorectal cancer detection using blink impression before and after the introduction of six blink features using a 2-minute online learning intervention. The study was approved by the Institutional Review Board of Ghent University Hospital (ONZ-2022–0488) and registered on ClinicalTrials.gov (NCT05699954). Informed consent was obtained from participating patients and endoscopists prior to the study.

### Phase 1: Selection of blink features

A comprehensive literature review was conducted in Medline, Embase, PubMed, and the Cochrane Library to identify gross morphological features of colorectal polyps associated with deep SMI ( **Supplementary Table 1** ). From the identified literature, six features were selected by author consensus (DJT, JA, LDs, LDb) based on three criteria: 1) strength of evidence linking the feature to deep SMI; 2) clinical relevance and frequency of observation; and 3) ease of identification by endoscopists of varying experience using white-light endoscopy without magnification or image enhancement. The selected features, collectively referred to as the six blink features were: 1) fold deformation, defined as convergence of ≥ 3 adjacent haustral folds towards, or interruption of an existing fold by, the lesion; 2) extra redness, defined as one or more foci of mucosal erythema on the lesion surface that are a deeper hue than the immediately-adjacent polyp tissue; 3) depression, defined as a clearly demarcated concavity or excavated area on the luminal surface of the polyp; 4) chicken-skin mucosa, defined as clusters of pale-yellow speckles in the surrounding mucosa (within ≈10 mm of the lesion margin); 5) ulceration, defined as a discrete mucosal defect exposing the subepithelial tissue on the lesion surface, usually covered by whitish fibrin/exudate (“white plaque”); and 6) spontaneous bleeding, defined as active oozing of blood from the lesion that is observed before any mechanical contact, irrigation or biopsy.

### Phase 2: Establishing expert consensus gold standard

Twenty images of LNPCPs were selected from a prospectively maintained tertiary referral center database to be broadly representative. The cases included 13 polyps without cancer, three with superficial SMI (< 1000 µm), and four with deep SMI (≥ 1000 µm). Four of these lesions had undergone prior manipulation (associated scarring) ( Table 1 , **Supplementary Table 2** ). Each image displayed the polyp in white-light endoscopy at a distance ≥ 10 mm, with adequate luminal distension and without magnification or virtual chromoendoscopy (Olympus, Tokyo, Japan).

**Table:** **Table 1** Demographic table.

**Colorectal Polyps,** (n = 20)	
**Size** , median mm (95%CI)	29.5 (22.3–36.7)
**Morphology,** n (%)	
non granular	12 (60)
granular	7 (35)
sessile serrated	1 (5)
**Paris classification,** n (%)	
Paris IIa	11 (55)
Paris Is component	7 (35)
Paris C component	2 (10)
**Location,** n (%)	
rectum	4 (20)
left colon	2 (10)
right colon	14 (70)
**Histopathology,** n (%)	
No Cancer	13 (65)
Superficial SMI	3 (15)
Deep SMI	4 (20)
**Participants,** (n = 165)	
**Profession,** n (%)	
Consultant gastroenterologist	105 (63.6)
Consultant surgeon	22 (13.3)
Trainee gastroenterologist	32 (19.4)
Trainee surgeon	3 (1.8)
Medical student	3 (1.8)
**Years in practice,** median [IQR]	6.5 [15.0]
**Lifetime colonoscopies** ≥ 1000 procedures, n (%)	94 (57.0)
**Lifetime pEMRs** ≥ 50 procedures, n (%)	21 (12.7)
**Continent of practice,** n (%)	
Europe	119 (72.1)
Africa	19 (11.5)
Oceania	12 (7.3)
Asia	15 (5.7)
Americas	6 (3.6)
Demographics of selected colorectal polyps and demographic and experiential profile of survey participants. CI, confidence interval; IQR, interquartile range; pEMR, piecemeal endoscopic mucosal resection; SMI, submucosal invasive cancer.	

Histopathological examination of all polyps was performed by an expert gastrointestinal pathologist (AH), blinded to endoscopic assessment. The pathologist determined presence and depth of cancer invasion, which served as the gold standard for blink (cancer vs no cancer). Only cases with SMI were classified as “cancer”; cases with “intramucosal cancer” were categorized as high-grade dysplasia and labelled “no cancer” for the purposes of this study.

Four authors (DJT, JA, LDs, LDb), blinded to histopathology, independently assessed the images through an online platform. For each image presence or absence of each blink feature was recorded. Thereafter, a structured consensus meeting was held to discuss discrepancies and establish a single gold standard for blink feature assignment.

### Phase 3: Validation of blink and blink features

The video outlines the blink feature concept with multiple video examples demonstrating identification of the different blink features in large non-pedunculated colorectal polyps with matched histopathology.Video 1

An online survey was developed using SurveyMonkey (California, United States) comprising four parts.

In the “Demographics and Experience” section, participants self-reported their role (consultant gastroenterologist, surgeon, trainee or medical student), years of experience, and procedural volumes (colonoscopies and piecemeal endoscopic mucosal resections [pEMRs]). General endoscopists were defined as those performing colonoscopy as part of routine clinical practice but without a recognized subspeciality focus in endoscopic imaging, optical diagnosis, or complex polyp resection (e.g., endoscopic mucosal resection/ESD).

In the blink Assessment (pre-intervention) section, participants viewed 20 LNPCP images in a random order and recorded their blink impression (presence of cancer: yes/no). Review was untimed.

During the educational intervention, participants were shown a 2-minute educational video ( Video 1 ) 29 , which introduced the six blink features with definitions and brief annotated examples distinct from the survey images.

During blink feature Identification (post-intervention), participants reviewed the same 20 images in a random order, recorded their blink impression and the presence or absence of each of the six blink features.

Participants were recruited via email through the mailing list of the Gastrointestinal Endoscopy Quality and Safety (GIEQs) Foundation (Ghent, Belgium), a not-for-profit educational initiative providing endoscopy training resources. An initial invitation was sent on November 9, 2022, followed by a reminder on November 22, 2022. The survey closed on November 28, 2022. Study images were not available on the GIEQs website, and therefore, had not been seen by participants prior to the study.

### Study endpoints

The primary endpoint was change in miss rate for recognizing histologically proven submucosal invasive cancer in LNPCPs using endoscopic imaging after exposure to six blink features. Secondary endpoints were change in specificity for recognizing cancer after blink-feature training, predictive value of each individual blink feature for discriminating cancer, association between blink-feature count and histologically confirmed SMI, interobserver agreement for presence/absence of each blink feature, and effect of endoscopist experience on diagnostic accuracy.

### Statistical analysis

All statistical analyses were performed using R statistical software (R Foundation, Indiana, United States) on anonymized data. Incomplete surveys were excluded from analysis.

Demographic data are presented as percentages, medians, and interquartile ranges. Sensitivity and specificity were estimated using generalized linear mixed models to account for multiple raters assessing the same polyps, with random effects for polyps and respondents 30 . Marginal estimates and pairwise contrasts were calculated using the emmeans package 31 . Stratified analyses assessed performance by experience level. A logistic mixed-effects model evaluated individual blink features as predictors of cancer, with performance assessed via five-fold cross-validation and receiver operating characteristic analysis. Full statistical methods are provided in Supplementary Methods.

## Results

### Study population

One hundred sixty-five endoscopists from 52 countries completed the online survey, generating 3,300 ratings of 20 LNPCPs before and after the intervention. Most were consultant gastroenterologists (105; 63.6%) or surgeons (22; 13.3%); 35 (21.2%) were trainees and the remainder medical students (3; 1.8%). Median self-reported colonoscopy experience was 6.5 years (IQR 15.0). More than half (94; 57.0%) had performed >1000 colonoscopies, and a smaller proportion (21; 12.7%) had completed at least 50 pEMRs. Europe predominated (119, 72.1%), but all continents were represented ( Table 1 , **Supplementary Table 3** ).

### Impact of exposure to six blink features on diagnostic performance

Performance before and after the 2-minute educational video was compared ( Table 2 ). At baseline, participants identified histologically proven cancer with 73.4% sensitivity (95% confidence interval [CI] 54.0–86.6) and 75.0% specificity (95% CI 61.5–84.9). After exposure to the concept of six blink features, sensitivity rose to 94.3% (95%CI 87.2–97.6), an absolute gain of 20.9% (95%CI 8.6–33.2; *P* < 0.001). The miss rate fell four-fold (from 26.6% to 5.7%) with a fall in specificity to 57.8% (95 %CI 41.8–72.3) and a rise in the false-alarm rate to 42.2% (95%CI 27.7–58.2).

**Table:** **Table 2** Overall and feature-specific diagnostic performance of the blink impression.

	Baseline	Post-intervention	Absolute Δ	*P*
**Global metrics**				
Sensitivity, % (95% CI)	73.4 (54.0–86.6)	94.3 (87.2–97.6)	+20.9 (8.6–33.2)	< 0.001
Miss-rate, % (95% CI)	26.6 (13.4–46.0)	5.7 (2.4–12.8)	-20.9 (-33.2 to -8.6)	< 0.001
Specificity, % (95% CI)	75.0 (61.5–84.9)	57.8 (41.8–72.3)	-17.2 (-22.9 to -11.4)	< 0.001
False-alarm rate, % (95% CI)	25.0 (15.1–38.5)	42.2 (27.7–58.2)	+17.2 (11.4–22.9)	< 0.001
**Individual cues**	** Sensitivity, % (95% CI) **	** Specificity, % (95% CI) **	** Adjusted OR (95% CI) **	***P***
Extra redness	72.0 (60.9–81.0)	72.9 (64.9–79.7)	3.66 (3.09–4.33)	< 0.001
Depression	64.4 (52.3–74.9)	71.1 (62.9–78.2)	3.06 (2.58–3.64)	< 0.001
Fold deformation	44.5 (32.7–56.9)	71.7 (63.5–78.7)	1.18 (0.99–1.40)	0.060
Spontaneous bleeding	27.1 (18.3–38.0)	94.2 (91.6–96.0)	3.92 (3.11–4.96)	< 0.001
Ulceration	21.3 (14.0–31.0)	92.5 (89.2–94.8)	1.11 (0.88–1.40)	0.359
Chicken-skin mucosa	17.2 (11.1–25.7)	89.1 (84.7–92.3)	1.03 (0.82–1.28)	0.822
The upper block presents accuracy metrics before and after the two-minute blink-feature educational intervention, including sensitivity, specificity, miss rate (1-sensitivity), and false alarm rate (i.e. false positive rate). The lower block displays per-blink-feature diagnostic indices and their independent associations with cancer. CI, confidence interval; OR, odds ratio.				

Subgroup analyses revealed the same pattern irrespective of seniority, colonoscopy count, or EMR experience ( **Supplementary Table 4, Supplementary Table 5, Supplementary Table 6** ).

### Number of blink features and histological invasion depth

**Fig. 2:**
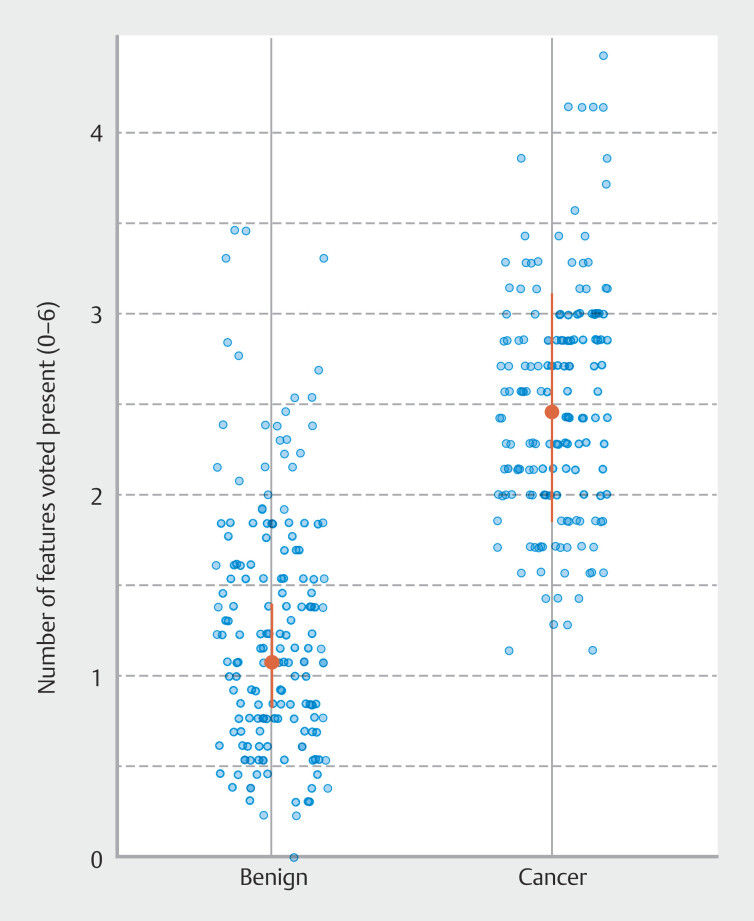
Correlation between blink-feature burden and histology. Dot-plot of rater level aggregated individual ratings (grey circles) showing the number of blink features identified per rater (ordinate, 0–6) stratified by final histology (benign vs cancer). Large red circles represent the group mean and red lines the 95% confidence intervals. The shift from left to the right in the figure illustrates the increased blink-feature burden in malignant lesions (linear-trend *P* < 0.001).

Mean number of blink features identified per polyp was plotted against final histology ( Fig. 2 ). Benign lesions yielded mean 1.08 (95% CI 0.82–1.40) features from participant ratings versus 2.46 (95% CI 1.85–3.12) in those with invasive cancer. Using a threshold of ≥ 2 features yielded 78.2% (95%CI 90.8–59.9) sensitivity and 70.5% (95%CI 80.8–57.3) specificity for invasive cancer, whereas ≥ 3 features were more specific (92.4% [95% CI 96.3–85.5]) but only modestly sensitive (47.6% [95% CI 69.2–27.2]) ( **Supplementary Table 7** ).

### Diagnostic yield of individual blink features

Extra redness was the most sensitive and specific (72.0%/72.9%) blink feature for invasive cancer among the respondents, followed by depression (64.4%/71.1%) and fold deformation (44.5%/71.7%). Conversely, spontaneous bleeding and ulceration were rare but telling signs, each occurring in < 30% of cancers yet with specificities > 92%. Chicken-skin mucosa was the least sensitive marker (17.2%) but had a high specificity of 89.1% ( Table 2 ).

**Fig. 3:**
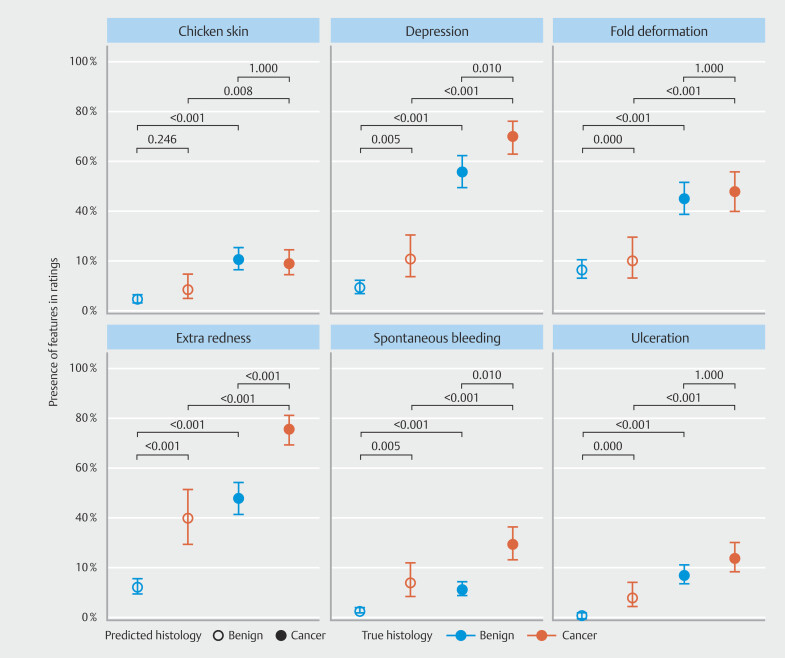
Percentage of specific blink features present in general endoscopist polyp ratings, stratified by true histological and predicted histologic status. Number of observations (predicted benign, true benign: 1189; predicted cancer, true benign: 956; predicted benign, true cancer: 137; predicted cancer, true cancer: 1018). Circles represent estimated marginal means; error bars indicate 95% confidence intervals.

Comparing post-intervention participant ratings (benign vs cancer) vs. histopathology, true-negatives (TNs) were the 1189 occasions a benign lesion was correctly dismissed, false-positives (FPs) the 956 ratings where benign lesions were actually cancer, false-negatives (FNs) the 137 ratings where cancers were missed, and true-positives (TPs) the 1018 ratings where cancers were correctly detected ( Fig. 3 ). Three patterns emerged: 1) extra redness, depression, fold deformation dominated correct cancer calls: They appeared in 76%, 70%, and 48% of TP ratings but in ≤ 21% of FN ratings; 2) specificity loss was driven by “over-calling” redness and fold deformation—both increased from 12% to 17% in TNs to about 40% to 45% in FP decisions; and 3) rare yet vivid signs—spontaneous bleeding and ulceration—were almost absent in TN (≤ 2.4%) but were observed significantly more in TPs (24%-29%) ( Table 3 ).

**Table:** **Table 3** Prevalence of each blink cue within diagnostic quadrants (3300 post-intervention ratings).

	True-negative (benign ↔ predicted benign) n= 1189	False-positive (benign ↔ predicted cancer) n= 956	False-negative (cancer ↔ predicted benign) n= 137	True-positive (cancer ↔ predicted cancer) n= 1018
Chicken-skin mucosa % (95% CI)	4.5 (3.2–6.3)	8.5 (4.7–15.0)	18.9 (14.4–24.5)	18.9 (14.4–24.5)
Depression % (95% CI)	9.3 (7.0–12.1)	20.9 (13.7–30.5)	69.9 (62.7–76.2)	69.9 (62.7–76.2)
Fold deformation % (95% CI)	16.5 (13.0–20.6)	20.2 (13.2–29.6)	47.9 (40.1–55.9)	47.9 (40.1–55.9)
Extra redness % (95% CI)	12.3 (9.5–15.7)	39.9 (29.5–51.4)	75.7 (69.2–81.2)	75.7 (69.2–81.2)
Spontaneous bleeding % (95% CI)	2.4 (1.6–3.7)	13.8 (8.4–21.9)	29.3 (23.1–36.4)	29.3 (23.1–36.4)
Ulceration % (95% CI)	0.9 (0.5–1.7)	7.9 (4.3–14.1)	23.7 (18.3–30.1)	23.7 (18.3–30.1)
Each cell shows the percentage of ratings in which the blink feature was marked present. Columns correspond to the four combinations of ground-truth histology and participant prediction. CI, confidence interval.				

### Multivariable modelling

The mixed-effects logistic model confirmed three features as independent predictors of general endoscopists detecting cancer within a given polyp ( Table 2 ): spontaneous bleeding (OR 3.92; 95% CI 3.11–4.96), extra redness(OR 3.66; 3.09–4.33) and depression (OR 3.06; 2.58–3.64). Fold deformation trended towards significance (OR 1.18; *P* = 0.060), whereas chicken-skin mucosa and ulceration were not significant after adjustment.

Model fit was excellent (area under the curve 0.79) ( **Supplementary Table 8, Supplementary Fig. 1** ) and the participant random effect collapsed to near-zero, indicating minimal unexplained between-rater heterogeneity once feature-level information was incorporated.

### Interobserver agreement

Raw post-intervention agreement for presence/absence of each feature ranged from 0.53 (depression) to 0.94 (spontaneous bleeding). Fleiss’ κ values were modest—0.14 for fold deformation and 0.51 for spontaneous bleeding. Intraclass correlation coefficients for the composite blink score varied from 0.45 to 0.75 across features ( **Supplementary Table 9** ).

## Discussion

This prospective, image-based interventional study demonstrates that the described six blink features fundamentally address the performance gap of general endoscopists—those who perform colonoscopy routinely but lack expertise in endoscopic imaging—in detecting cancer within colorectal polyps. After a 2-minute online introduction to the blink-feature concept, the cancer miss rate fell four-fold. Importantly, the included images were taken using standard white-light endoscopy without magnification or virtual chromoendoscopy, making these improvements immediately accessible to the majority of endoscopists who lack advanced imaging expertise. The study relevance lies not in enhancing expert performance—specialists in endoscopic imaging have already developed sophisticated unconscious blink pattern recognition—but in democratizing cancer detection for the broader endoscopic community.

### Primary endpoint: Large, uniform gain in sensitivity

Breaking down the first impression of cancer into six blink features delivered a 21% absolute rise in sensitivity among general endoscopists. The effect was consistent across consultants, trainees, and surgeons. The magnitude of this effect—reducing the miss rate from 27% to 6%—represents a substantial improvement in this controlled setting. If similar gains in the live environment could be made may prevent up to one in five cancers from being inappropriately managed endoscopically. The price of this heightened vigilance was a 17% fall in specificity (from 75% to 58%). This trade-off is familiar in screening medicine: More disease is captured at the expense of more benign lesions being labelled suspicious 32 .

Clinical implications of this sensitivity-specificity balance require careful consideration within the colorectal polyp management pathway. Over-calling benign polyps should trigger established safety nets—multidisciplinary team review or referral for expert optical diagnosis—that effectively mitigate potential harm 33 . In contrast, the consequences of missing cancer are substantial and potentially irreversible: attempted pEMR with positive margins, perforation and tumor seeding, and, delay to definitive oncological therapy. Given these asymmetric consequences, the resultant sensitivity-specificity balance appears not only acceptable but potentially desirable for those endoscopists performing frontline assessments.

### Secondary endpoints

To understand why participants succeeded or failed after training, we mapped every rating onto a 2×2 matrix of ground-truth histology (benign/cancer) versus participant verdict (benign/cancer). Three distinct patterns emerged from this analysis: 1) blink features that secure correct cancer calls; 2) features that erode specificity; and 3) rare but powerful signs.

When cancers were correctly identified, extra redness, depression, and fold deformation were the most common cues recognized by participants. These same features showed markedly different prevalence in false-negative decisions, suggesting that failure to recognize these signs—rather than their absence—drove most misclassifications. This pattern implies that further refinement of teaching materials could focus on threshold recognition: at what point does mild erythema become "extra redness," and when does surface irregularity constitute a true "depression".

False-positive escalation of benign polyps was largely driven by over-calling extra redness and fold deformation. The high false-positive rate for fold deformation may reflect a specific clinical scenario: Scarring from previous incomplete resection can produce architectural distortion mimicking invasive growth. Teaching, therefore, must refine when these two signs truly imply invasion versus benign fibrosis, potentially by including number of folds converging within the polyp or combining with high specificity features. Chicken-skin mucosa added little independent information once the other cues were known in this dataset, suggesting potential redundancy in the current six-item framework. Future validation may determine whether omitting this feature could further minimize cognitive load.

Spontaneous bleeding and ulceration remained almost absent in TNs yet increased dramatically in TPs, explaining their very high specificities and strong adjusted ORs in the multivariate model. These "high-value" signs function as near-pathognomonic markers of invasion when present, though their relative rarity in the dataset limited their contribution to overall sensitivity.

Together, these patterns both help us understand how cancer decisions are made by general endoscopists and hint at a targeted refinement for the next training iteration: Raise awareness that there should be a higher diagnostic threshold/information for extra redness and fold deformation while reinforcing that spontaneous bleeding and ulceration, although uncommon, are high-yield indicators of malignancy. Seen differently, these are precisely the situations where expert assessment is important. These features can identify areas of polyps which can be carefully interrogated with magnification and chromoendoscopy for pit/vascular pattern analysis to reach a precise diagnosis. However, the lesion must first be detected and escalated to the expert, and so, both strategies and structured communication between colleagues, therefore, are essential.

The multivariate model random-participant variance collapsed towards zero, indicating that once feature use was accounted for, little unexplained heterogeneity remained between endoscopists. Such a pattern strongly supports this structured training approach over experiential learning alone.

### Interobserver agreement

Despite increased sensitivity using the framework, Fleiss' κ for most features stayed in the slight-to-fair range, with only spontaneous bleeding achieving moderate agreement. This persistent interobserver variability is not unique to our study 34 . Importantly, however, despite modest feature-level agreement, the overall framework still produced substantial improvements in overall diagnostic performance, suggesting that its value lies in its overall outcome (number of features) rather than perfect concordance on individual features.

### Experience still helps, but structure helps more

Among participants, those with extensive colonoscopy experience posted the highest post-training sensitivity, yet trainees showed the greatest absolute gain, underscoring that explicit deconstruction of a problem can compress learning curves. This differential improvement pattern reveals an important distinction: Experience in performing colonoscopy does not automatically confer expertise in endoscopic imaging. Many experienced gastroenterologists lack formal training in optical diagnosis, whereas conversely, some junior endoscopists may have received structured imaging education. The blink framework bridges this specific skill gap by making a certain amount of tacit imaging knowledge explicit, allowing general endoscopists to appropriate pattern recognition strategies typically reserved for imaging specialists.

That said, specificity dropped equally across all experience strata, reinforcing that if considering improving upon these results in the future the challenge lies not in experience per se but in calibrating appropriate thresholds for suspicious features and criteria for expert referral via multidisciplinary discussion.

### Grounding findings in learning theory

According to dual-process models of cognition 9 , experts use fast, pattern-recognition "System 1" heuristics, whereas novices rely on slower, analytic "System 2" reasoning. Our data suggest that explicitly labelling key visual patterns (introducing blink features framework) allows general endoscopists to appropriate elements of System 1 thinking almost immediately—a concept validated in parallel fields of medicine 10 11 . First, it reduces intrinsic cognitive load by chunking complex visual information into six discrete, memorable features. Second, it minimizes extraneous load by providing a consistent evaluation sequence. Third, it converts vague intuitions ("this looks worrying") into explicit, teachable schemas that can be rehearsed and automated through deliberate practice. This scaffolding is particularly valuable in endoscopy, where real-time decision-making under the cognitive pressure of a complex motor skill often precludes extensive deliberation.

### Strengths and limitations

Strengths of this study include the large globally diverse cohort, a consensual expert gold standard with forced consensus methodology, pre-post design and mixed-effects statistics that appropriately account for clustering within participants. The image quality—obtained from standard-definition endoscopes with good preparation—likely represents real-life conditions for visualization.

Several limitations warrant mention. First, our 20-image set was enriched for cancer (~35 %), potentially boosting sensitivity relative to everyday practice; repeating the identical images after the learning intervention also risks a recall effect (somewhat mitigated by randomization). Second, the small sample restricts statistical power and makes κ estimates imprecise. Third, still photographs omit dynamic cues (bleeding on contact, fold deformation during peristalsis) and the cognitive load of live colonoscopy, so translation to real-time performance remains unproven. In routine practice, suboptimal preparation or difficult insertion could reduce their diagnostic performance. Fourth, the study measures diagnostic accuracy only; we did not track downstream outcomes to show fewer interval cancers or avoided surgeries, nor did we quantify the additional referrals generated by the specificity drop. Fifth, participants were recruited through the GIEQs mailing list, which likely represents a motivated and education-oriented group of endoscopists; this may have had bi-directional effects on diagnostic performance. Sixth, the survey also did not include clinical endoscopists as a distinct professional category, indicating a missed opportunity. Furthermore, it included a small number of medical students whose limited experience may have influenced baseline and learning-effect estimates. Finally, our binary benign-vs-cancer framework does not accommodate the clinical gray zone of indeterminate lesions.

### Future directions: Blink first, then look closer

We propose a two-tier implementation pathway integrating rapid screening with detailed analysis. When an LNPCP is identified, the operator pauses the endoscopic image and performs a rapid assessment of the six blink features. If two or more are present, this triggers mandatory escalation. In interventional endoscopy settings, this means proceeding to (virtual) chromoendoscopy and magnification to evaluate established criteria (e.g. vascular/pit pattern analysis). In screening examinations, high-quality images and video should be captured for multidisciplinary team review. This approach respects efficiency demands while providing a safety net against the documented false-positive tendency.

For those seeking to optimize their specificity using this framework, threshold refinement through borderline case libraries—particularly for redness and fold deformation—may further enhance performance. However, the core value proposition remains sensitivity improvement for general endoscopists. The six blink features also constitute human-interpretable labels that could seed explainable artificial intelligence algorithms, enabling real-time decision support that transparently indicates which features triggered concern.

## Conclusions

A concise six-item blink framework reduced the miss rate for invasive cancer in LNPCP still images among general endoscopists four-fold, while revealing precisely which visual cues drive both successful detection and false alarms. Embedding this structured approach into training curricula, coupled with selective use of confirmatory imaging for screen-positive lesions, could offer an evidence-based pathway to reduce missed cancers while managing the increased FP rate through existing clinical safeguards. Further validation in live endoscopy settings will determine whether these gains in still-image interpretation translate to meaningful improvements in real-world patient outcomes.

## Prior preprint disclosure

An earlier version of this manuscript was made available as a preprint 35 .

## Supplementary Material

Supplementary Material Zusatzmaterial

## References

[1] Zauber AG Winawer SJ O'Brien MJ Colonoscopic polypectomy and long-term prevention of colorectal-cancer deaths N Engl J Med 2012 366 687 696 10.1056/NEJMoa1100370 22356322 PMC3322371

[2] Moss A Bourke MJ Williams SJ Endoscopic mucosal resection outcomes and prediction of submucosal cancer from advanced colonic mucosal neoplasia Gastroenterology 2011 140 1909 1918 21392504 10.1053/j.gastro.2011.02.062

[3] Jayanna M Burgess NG Singh R Cost analysis of endoscopic mucosal resection vs surgery for large laterally spreading colorectal lesions Clin Gastroenterol Hepatology 2016 14 271 272 10.1016/j.cgh.2015.08.037 26364679

[4] Keswani RN Law R Ciolino JD Adverse events after surgery for nonmalignant colon polyps are common and associated with increased length of stay and costs Gastrointest Endosc 2016 84 296 3.03E293 26828760 10.1016/j.gie.2016.01.048

[5] Saito Y Sakamoto T Dekker E First report from the International Evaluation of Endoscopic classification Japan NBI Expert Team: International multicenter web trial Dig Endosc 2024 36 591 599 37702082 10.1111/den.14682

[6] Dekker E Houwen B Puig I Curriculum for optical diagnosis training in Europe: European Society of Gastrointestinal Endoscopy (ESGE) Position Statement Endoscopy 2020 52 899 923 10.1055/a-1231-5123 32882737

[7] Meulen LWT van de Wetering AJP Debeuf MPH Optical diagnosis of T1 CRCs and treatment consequences in the Dutch CRC screening programme Gut 2020 69 2049 2051 10.1136/gutjnl-2019-320403 31937551

[8] Meulen LWT Haasnoot KJC Vlug MS Effect of optical diagnosis training on recognition and treatment of submucosal invasive colorectal cancer in community hospitals: a prospective multicenter intervention study Endoscopy 2024 56 770 779 10.1055/a-2313-4996 38657659 PMC11436291

[9] Kahneman D Thinking, fast and slow. 1st ed New York Farrar, Straus and Giroux 2011

[10] Raat EM Farr I Wolfe JM Comparable prediction of breast cancer risk from a glimpse or a first impression of a mammogram Cogn Res Princ Implic 2021 6 72 10.1186/s41235-021-00339-5 34743266 PMC8572261

[11] Evans KK Georgian-Smith D Tambouret R The gist of the abnormal: above-chance medical decision making in the blink of an eye Psychon Bull Rev 2013 20 1170 1175 10.3758/s13423-013-0459-3 23771399 PMC3851597

[12] Puig I Mármol C Bustamante M Endoscopic imaging techniques for detecting early colorectal cancer Curr Opin Gastroenterol 2019 35 432 439 10.1097/MOG.0000000000000570 31246596

[13] Bugajski M Kaminski MF Orlowska J Suspicious macroscopic features of small malignant colorectal polyps Scand J Gastroenterol 2015 50 1261 1267 10.3109/00365521.2015.1024280 25865832

[14] Guan J Zhao R Zhang X Chicken skin mucosa surrounding adult colorectal adenomas is a risk factor for carcinogenesis Am J Clin Oncol 2012 35 527 532 10.1097/COC.0b013e31821dedf7 21654311

[15] Horie H Togashi K Kawamura YJ Colonoscopic stigmata of 1 mm or deeper submucosal invasion in colorectal cancer Dis Colon Rectum 2008 51 1529 1534 10.1007/s10350-008-9263-y 18592315

[16] Ikehara H Saito Y Matsuda T Diagnosis of depth of invasion for early colorectal cancer using magnifying colonoscopy J Gastroenterol Hepatol 2010 25 905 912 10.1111/j.1440-1746.2010.06275.x 20546444

[17] Jang HW Park SJ Cheon JH Does magnifying narrow-band imaging or magnifying chromoendoscopy help experienced endoscopists assess invasion depth of large sessile and flat polyps? Dig Dis Sci 2014 59 1520 1528 10.1007/s10620-014-3090-x 24839918

[18] Kudo S Hirota S Nakajima T Colorectal tumours and pit pattern J Clin Path 1994 47 880 885 10.1136/jcp.47.10.880 7962600 PMC502170

[19] Kudo S Kashida H Tamura T Colonoscopic diagnosis and management of nonpolypoid early colorectal cancer World J Surg 2000 24 1081 1090 10.1007/s002680010154 11036286

[20] Lee YM Song KH Koo HS Colonic chicken skin mucosa surrounding colon polyps is an endoscopic predictive marker for colonic neoplastic polyps Gut Liver 2022 16 754 763 10.5009/gnl210271 35000932 PMC9474497

[21] Matsuda T Saito Y Hotta K Prevalence and clinicopathological features of nonpolypoid colorectal neoplasms: should we pay more attention to identifying flat and depressed lesions? Dig Endosc 2010 22 S57 S62 20590774 10.1111/j.1443-1661.2010.00967.x

[22] Misawa M Kudo SE Wada Y Magnifying narrow-band imaging of surface patterns for diagnosing colorectal cancer Oncol Rep 2013 30 350 356 10.3892/or.2013.2471 23673484

[23] Nowicki MJ Bishop PR Subramony C Colonic chicken-skin mucosa in children with polyps is not a preneoplastic lesion J Pediatr Gastroenterol Nutr 2005 41 600 606 16254516 10.1097/01.mpg.0000179658.09210.b6

[24] Puig I López-Cerón M Arnau A Accuracy of the narrow-band imaging international colorectal endoscopic classification system in identification of deep invasion in colorectal polyps Gastroenterology 2019 156 75 87 10.1053/j.gastro.2018.10.004 30296432

[25] Saito Y Fujii T Kondo H Endoscopic treatment for laterally spreading tumors in the colon Endoscopy 2001 33 682 686 10.1055/s-2001-16213 11490384

[26] Saitoh Y Obara T Watari J Invasion depth diagnosis of depressed type early colorectal cancers by combined use of videoendoscopy and chromoendoscopy Gastrointest Endosc 1998 48 362 370 10.1016/s0016-5107(98)70004-5 9786107

[27] Shatz BA Weinstock LB Thyssen EP Colonic chicken skin mucosa: an endoscopic and histological abnormality adjacent to colonic neoplasms Am J Gastroenterol 1998 93 623 627 10.1111/j.1572-0241.1998.177_b.x 9576459

[28] Uno Y Munakata A Endoscopic and histologic correlates of colorectal polyp bleeding Gastrointest Endosc 1995 41 460 467 10.1016/s0016-5107(05)80004-5 7615224

[29] Tate DJ Introduction to 6 blink features-learning tool https://vimeo.com/768168090

[30] Bates D Mächler M Bolker B Fitting linear mixed-effects models using lme4 J Stat Software 2015 67 1 48

[31] Lenth RV Piaskowski J emmeans: Estimated Marginal Means, aka Least-Squares Means. R package version 1.11.2. 2025 https://CRAN.R-project.org/package=emmeans

[32] Shaughnessy AF Clinical epidemiology: A basic science for clinical medicine BMJ 2007 335 777

[33] Di Fabio F Jitsumura M Longstaff L Management of significant polyp and early colorectal cancer is optimized by implementation of a dedicated multidisciplinary team meeting: lessons learned from the United Kingdom National Program Dis Colon Rectum 2022 65 654 662 34840306 10.1097/DCR.0000000000002199

[34] van Doorn SC Hazewinkel Y East JE Polyp morphology: an interobserver evaluation for the Paris classification among international experts Am J Gastroenterol 2015 110 180 187 25331346 10.1038/ajg.2014.326

[35] Debels LK Tate DJ Teaching six ‘blink’ features reduces general endoscopists’ cancer miss-rate in image-based assessment of large colorectal polyps Research Square [Preprint] 2026 10.21203/rs.3.rs-8553404/v1 PMC13289961 42344381

