# Supplementary material for: Teaching Six “blink” features reduces general endoscopist cancer miss rate in image-based assessment of large colorectal polyps
Source: Endosc Int Open. 2026 May 29;14:a28684812. doi: 10.1055/a-2868-4812 (PMC13289961; doi:10.1055/a-2868-4812)
Supplement: Supplementary file 2 — Supplementary Material [file 10-1055-a-2868-4812_28768172.pdf]

Narrative Review: The six blink features for identifying deep submucosal invasion in colorectal polyps

Introduction

This narrative review synthesizes available evidence supporting six morphological features - termed "Blink features" - for identifying deep submucosal invasion (SMI) in colorectal polyps using white-light endoscopy without magnification or virtual chromoendoscopy. These features were selected through expert consensus based on their ease of identification and clinical utility in routine endoscopic practice.

Methods

A comprehensive literature search was conducted in Medline, Embase, PubMed, and the Cochrane Library to identify gross morphological features of colorectal polyps associated with deep submucosal invasion. Search terms included combinations of "colorectal polyps," "submucosal invasion," "endoscopic features," "morphological characteristics," and specific feature descriptors. From the identified literature, six features were selected by author consensus (DJT, JA, LDs, LDb) based on three criteria: (1) strength of evidence linking the feature to deep SMI, (2) clinical relevance and frequency of observation, and (3) ease of identification by endoscopists of varying experience using white-light endoscopy without magnification or image enhancement. The selected features were evaluated against the supporting literature to develop evidence-based definitions.

Morphological features: Definitions and supporting evidence

1. Fold Deformation

Definition: *"Convergence of ≥ 3 adjacent haustral folds towards, or interruption of an existing fold by, the lesion."*

The specificity of requiring ≥3 converging folds emerged from Ikehara et al. [1], who demonstrated this pattern as an independent marker of deep invasion with an odds ratio of 3.4. Uraoka et al. [2] validated "fold convergency towards the tumour" as one of eight macroscopic criteria for submucosal invasion in their endoscopic mucosal resection (EMR) series. Additional support comes from Jang et al. [3], who assessed fold changes in invasion depth determination, while Saitoh et al. [4] utilized combined videoendoscopy and chromoendoscopy to evaluate fold deformation patterns. Kudo et

al. [5] and Saito et al. [6] further established fold convergence as a reliable indicator, with Puig et al. [7] incorporating this feature into the NICE classification system for deep invasion assessment.

2. Extra Redness

Definition: *"One or more foci of mucosal erythema on the lesion surface that are a deeper hue than the immediately-adjacent polyp tissue."*

Uraoka et al. [2] prospectively scored "prominent surface redness" in their laterally spreading tumor (LST) dataset, demonstrating its linkage to higher submucosal invasion risk. Crucially, Uno and Munakata [8] established the biological basis for this feature, correlating red color tone with surface micro-erosion that precedes bleeding, confirming that redness represents biologically active surface change rather than merely a visual phenomenon. This pathophysiological understanding strengthens the feature's validity as a predictor of invasive behavior.

3. Depression

Definition: *"A clearly demarcated concavity or excavated area on the luminal surface of the polyp."*

Depression demonstrated the strongest evidence base with both "demarcated depressed area" and "deep depression" serving as independent invasion predictors across multiple series. Uraoka et al. [2] and the e-T2 scoring system developed by Ikehara et al. [1] confirmed its predictive value. Supporting evidence includes Jang et al. [3] and Puig et al. [7] who incorporated depression into classification criteria. Saito et al. [6] identified depression in laterally spreading tumors requiring resection, while Bugajski et al. [9] found it in small malignant polyps. Saitoh et al. [4] combined videoendoscopy and chromoendoscopy to evaluate depressed cancers. Matsuda et al. [10] emphasized the clinical importance of identifying depressed lesions, with Horie et al. [11] confirming depression as a colonoscopic stigmata of ≥1mm invasion. Misawa et al. [12] utilized narrow-band imaging to further validate depressed surface patterns as markers of invasion.

4. Chicken-Skin Mucosa

Definition: *"Clusters of pale-yellow speckles in the surrounding mucosa (within ~10 mm of the lesion margin)."*

Shatz et al. [13] first described this "pale yellow-speckled mucosa" adjacent to neoplasms, coining the term "chicken-skin mucosa" - a finding that established this

feature's recognition in the endoscopic literature. Uraoka et al. [2] prospectively tabulated "chicken-skin mucosa around the tumour" in their EMR cohort, validating its clinical significance. The spatial specification of within approximately 10 mm of the lesion margin provides crucial guidance for endoscopic assessment. Supporting studies include Jang et al. [3], Lee et al. [14] who confirmed it as a predictive marker for neoplastic polyps, Nowicki et al. [15] who investigated its significance in pediatric populations, Guan et al. [16] who established it as a carcinogenesis risk factor, and Bugajski et al. [9] who incorporated it into suspicious feature assessments. Puig et al. [7] also noted perilesional changes in their classification system.

5. Ulceration

Definition: *"A discrete mucosal defect exposing the subepithelial tissue on the lesion surface, usually covered by whitish fibrin/exudate ('white plaque')."*

The e-T2 scoring system by Ikehara et al. [1] treated "erosion or white plaque" - synonymous with shallow ulcer covered by fibrin - as one of five independent signs of T2 cancer. Puig et al. [7] identified ulceration as an accuracy-limiting co-factor in their NBI deep-invasion study, highlighting its importance in assessment algorithms. The characteristic white plaque appearance provides a clear visual marker supported by Uno et al. [8], who established histologic correlates of surface erosion. Horie et al. [11] confirmed ulceration as a colonoscopic stigmata of ≥1mm submucosal invasion, while Kudo et al. [17] incorporated surface erosion patterns into their pit pattern classification system.

6. Spontaneous Bleeding

Definition: *"Active oozing of blood from the lesion that is observed before any mechanical contact, irrigation or biopsy."*

The critical distinction of bleeding occurring before any manipulation was emphasized by experienced endoscopists in Jang et al. [3], who included surface bleeding among conventional signs evaluated when no manipulation had yet been performed. Uno and Munakata [8] provided the pathological basis for 'spontaneous' bleeding, demonstrating that it correlates with surface micro-erosion and occurs independently of instrument contact. This biological validation distinguishes true spontaneous bleeding from procedure-induced hemorrhage. Additional support comes from Horie et al. [11] and Bugajski et al. [9], who confirmed spontaneous bleeding as a stigmata of deep invasion.

Clinical application and synthesis

The definitions provide precise criteria for clinical application. Key quantitative thresholds include:

- Fold deformation requiring ≥3 converging folds (OR 3.4 for deep invasion)
- Chicken-skin mucosa identified within ~10 mm of lesion margins
- Spontaneous bleeding observed before any mechanical contact

These features integrate multiple assessment dimensions: structural alterations (fold deformation, depression), vascular/inflammatory changes (extra redness, spontaneous bleeding), tissue disruption (ulceration), and perilesional markers (chicken-skin mucosa). The biological correlations established by Uno and Munakata [8] for redness and bleeding provide mechanistic understanding beyond empirical observation.

The evidence base combines prospective cohort studies (Uraoka et al. [2]), validation of scoring systems (e-T2 score by Ikehara et al. [1]), and pathophysiological correlation studies (Uno and Munakata [8]). The features were validated across different lesion types including laterally spreading tumors, small malignant polyps, and depressed lesions, enhancing generalizability.

Limitations

This narrative review has several limitations. First, the literature selection was based on expert consensus rather than exhaustive systematic searching, potentially missing relevant studies. Second, formal quality assessment of included studies was not performed, and the relative strength of evidence varies across features. Third, the definitions were developed through consensus rather than prospective validation. Finally, inter-observer variability in identifying these features has not been systematically evaluated across all six criteria prior to the current study.

Future directions

Several areas warrant further investigation:

1. Prospective validation of all six Blink features as a combined assessment tool
2. Interobserver agreement studies among endoscopists of varying experience levels

3. Development of standardized training modules for feature recognition
4. Integration with advanced imaging technologies (NBI, chromoendoscopy) to enhance accuracy
5. Cost-effectiveness analysis of using these features for clinical decision-making
6. Development of artificial intelligence algorithms for automated feature detection
7. Evaluation of feature performance across different ethnic populations and geographic regions

Conclusions

This narrative review establishes six precisely defined Blink features as evidence-based morphological indicators of deep submucosal invasion in colorectal polyps. The definitions provide a standardized framework for endoscopic assessment that can be applied using white-light endoscopy without requiring advanced imaging modalities. While individual features demonstrate varying levels of evidence, their combined application offers endoscopists of varying experience levels a practical tool for risk stratification and clinical decision-making in the management of colorectal polyps

Supplementary Table 1 Overview of literature-derived gross morphological features associated with deep submucosal invasive colorectal cancer, reviewed to inform the development of the Blink framework.

#	Proposed definition	Key supportive literature
1 Fold deformation	"Convergence of ≥ 3 adjacent haustral folds towards, or interruption of an existing fold by, the lesion."	• Ikehara et al. counted ≥3 converging folds as an independent marker of deep invasion (odds ratio 3.4) [1]<br>• Uraoka's EMR series classified "fold convergency towards the tumour" as one of eight macroscopic criteria for submucosal invasion [2]
2 Extra redness	"One or more foci of mucosal erythema on the lesion surface that are a deeper hue than the immediately-adjacent polyp tissue."	• "Prominent surface redness" was prospectively scored in Uraoka's LST data set and linked to higher SM-invasion risk [2]<br>• Uno & Munakata correlated a red colour tone with surface micro-erosion that precedes bleeding, confirming that redness represents biologically active surface change. [8]
3 Depression	"A clearly demarcated concavity or excavated area on the luminal surface of the polyp."	• Both "demarcated depressed area" and "deep depression" were independent invasion predictors in multiple series (Uraoka; Koyama e-T2 score). [1, 2]
4 Chicken-skin mucosa	"Clusters of pale-yellow speckles in the surrounding mucosa (within ≈10 mm of the lesion margin)."	• Shatz et al. first described the "pale yellow-speckled mucosa" adjacent to neoplasms, coining the term "chicken-skin mucosa". [13]<br>• The same appearance was prospectively tabulated as "chicken-skin mucosa around the tumour" in Uraoka's EMR cohort. [2]
5 Ulceration	"A discrete mucosal defect exposing the subepithelial tissue on the lesion surface, usually covered by whitish fibrin/exudate ('white plaque')."	• The e-T2 score treated "erosion or white plaque" (a synonym for shallow ulcer covered by fibrin) as one of five independent signs of T2 cancer. [1]<br>• Ulceration was also an accuracy-limiting co-factor in Puig's NBI-deep-invasion study. [7]

6 Spontaneous bleeding	"Active oozing of blood from the lesion that is observed **before** any mechanical contact, irrigation or biopsy."	• Uno & Munakata demonstrated that bleeding correlates with surface micro-erosion and occurs independently of instrument contact, providing the pathological basis for 'spontaneous' bleeding. [8]<br>• Experienced endoscopists in Jang et al. included surface bleeding among the conventional signs evaluated when no manipulation had yet been performed. [3]

*Only the highest-quality or most frequently cited studies are highlighted for each feature.

Methods

Statistical Analyses

All statistical analyses were performed using R statistical software (R Foundation for Statistical Computing, Vienna, Austria). Surveys with incomplete responses were excluded from the analysis to ensure data completeness.

Descriptive statistics

Demographic and experience data are presented as percentages for categorical variables, and medians with interquartile ranges (25th-75th percentiles) for continuous variables, reflecting the non-normal distribution of experience metrics.

Primary analysis: Generalized linear mixed models

Given the hierarchical study design, with multiple raters assessing the same set of 20 polyps, we employed generalized linear mixed models (GLMMs) to appropriately handle the clustered data structure. These models analyzed individual respondent predictions of cancer (binary outcome) as a function of:

- Ground truth histology (cancer vs. benign)
- Timepoint (pre- vs. post-intervention)
- Their interaction

The model specification included:

- Random intercepts for polyps (accounting for inherent differences in polyp difficulty)
- Random intercepts for respondents (accounting for baseline differences in diagnostic ability)
- Random slopes for respondents across timepoints (allowing for differential learning effects)

This approach accounts for the non-independence of observations arising from repeated measures across both raters and polyps. Models were fitted using the lme4 package in R [18], employing maximum likelihood estimation with adaptive Gauss-Hermite quadrature for improved accuracy in binary outcome models.

Estimation of diagnostic performance metrics

Marginal probabilities, sensitivity, and specificity were estimated from the fitted models using the emmeans package [19]. This approach provides model-based estimates that appropriately account for the random effects structure. Pairwise contrasts between

pre- and post-intervention timepoints were calculated with Wald-type confidence intervals and p-values adjusted for multiple comparisons where appropriate.

Stratified dnalyses

To assess whether the intervention effect varied by experience level, we fitted stratified models by:

• Respondent grade (consultant vs. trainee vs. surgeon)

• Colonoscopy experience (>1000 vs. ≤1000 procedures)

• EMR experience (≥50 vs. <50 procedures)

Each stratified model maintained the same random effects structure as the primary analysis.

Multivariable modeling of blink features

To identify which Blink features independently predicted cancer detection, we constructed a logistic mixed-effects model with:

• Outcome: Cancer status (binary)

• Fixed effects: Presence/absence of each of the six Blink features

• Random effect: Random intercept for respondents (accounting for inter-rater variability)

Model validation and performance assessment

Model performance was rigorously evaluated using 5-fold cross-validation to prevent overfitting:

1. The dataset was randomly partitioned into 5 equal folds

2. For each fold:

   – The model was trained on 4 folds (80% of data)

   – Optimal classification threshold was determined using Youden's Index (maximizing sensitivity + specificity - 1) on the training set

   – Performance metrics (AUC, accuracy, sensitivity, specificity) were calculated on the held-out test fold (20% of data)

3. Final performance was reported as the mean across all 5 folds with 95% confidence intervals

Model diagnostics

Discrimination was assessed using receiver operating characteristic (ROC) curves, with the area under the curve (AUC) providing a summary measure of discriminative ability. Calibration was evaluated using calibration plots comparing predicted

probabilities against observed frequencies within decile groups of predicted risk. These visualizations were based on predicted probabilities from the final model fitted to the complete dataset.

Interobserver agreement

Agreement between raters was assessed using:

- Raw agreement proportions for binary features
- Fleiss' kappa for multi-rater agreement on categorical outcomes
- Intraclass correlation coefficients (ICC(2,1) two-way random) for the continuous Blink score

All statistical tests were two-sided with significance set at $P < 0.05$.

Results Tables

Supplementary Table 2 Characteristics of the colorectal polyps included in the survey.

Polyp number	Blink impression expert	Blink features expert	Number of Blink features expert	Histopathology	Previous manipulation (recurrence /residual)
1	-	Extra redness	1	No cancer	-
2	+	Fold deformation<br>Extra redness<br>Chicken-skin mucosa	3	Deep	-
3	+	Fold deformation<br>Extra redness<br>Chicken-skin mucosa	3	Deep	-
4	+	Spontaneous bleeding<br>Fold deformation<br>Extra redness	3	Deep	-
5	+	Extra redness<br>depression	2	Superficial	-
6	+	Depression<br>Fold deformation<br>Extra redness<br>Chicken-skin mucosa	4	Superficial	-
7	-	Chicken-skin mucosa<br>Depression<br>Extra redness	2	No cancer	-
8	+	Fold deformation<br>Extra redness<br>Spontaneous bleeding	3	Deep	-
9	-	Fold deformation	2	No cancer	+
10	+	Extra redness	3	Superficial	+

		Fold deformation<br>Depression		
11	-		No cancer	+
12	-		No cancer	+
13	-		No cancer	-
14	-		No cancer	-
15	-		No cancer	-
16	-		No cancer	-
17	-		No cancer	-
18	-		No cancer	-
19	-		No cancer	-
20	-		No cancer	-

Superficial: Superficially invasive submucosal invasive cancer (<1000 µm); deep: deeply invasive submucosal invasive cancer(≥1000 µm). + feature present, - feature absent.

Supplementary table 3 Recruitment and response process of the invited participants.

3187 (100%) invited participants	
262 (8.2%) responded	Exclusion of 2925 (91.8%) non-responders
165 (5.2%) completed	Exclusion of 97 (3.0%) incomplete responses
= 6600 observations (2x3300 polyps)	

Supplementary Table 4 Sensitivity and specificity for detection of cancer in colorectal polyps, measured before (baseline) and after (post-intervention) the two-minute Blink-feature educational intervention, stratified by participant profession.

Profession	Sensitivity baseline	Sensitivity post-intervention	Specificity baseline	Specificity post-intervention
**Consultant gastroenterologist % (95% CI)**	76.0 (56.5-88.5)	95.0 (88.2-98.0)	78.3 (65.1-87.5)	62.7 (45.9-76.9)
**Consultant surgeon % (95% CI)**	61.6 (36.7-81.6)	87.9 (69.3-95.9)	69.9 (52.2-83.2)	56.7 (34.8-76.3)
**Trainee gastroenterologist**% (95% CI)	72.6 (51.6-86.7)	96.6 (90.5-98.8)	69.1 (53.1-81.6)	41.2 (24.6-60.1)
**Trainee surgeon % (95% CI)**	77.5 (40.2-94.7)	91.4 (60.5-98.7)	74.1 (43.3-91.5)	64.9 (32.6-87.6)
**Medical student % (95% CI)**	70.7 (21.9-95.4)	88.3 (38.1-98.9)	72.6 (31.7-93.8)	65.5 (20.5-93.3)

CI, confidence interval.

Supplementary Table 5 Sensitivity and specificity for detection of cancer in colorectal polyps, measured before (baseline) and after (post-intervention) the two-minute Blink-feature educational intervention, stratified by participant colonoscopy experience.

Colonoscopy experience	Sensitivity baseline	Sensitivity post-intervention	Specificity baseline	Specificity post-intervention
**0-50 % (95% CI)**	66.0 (39.3-85.3)	91.0 (72.5-97.5)	65.1 (44.3-81.3)	36.0 (16.4-61.6)
**51-200 % (95% CI)**	83.4 (59.8-94.5)	97.7 (90.5-99.5)	64.9 (42.2-82.4)	41.8 (19.9-67.5)
**201-400 % (95% CI)**	57.6 (33.3-78.7)	88.8 (70.2-96.4)	70.4 (52.8-83.5)	45.6 (25.6-67.2)
**401-999 % (95% CI)**	69.0 (46.8-85.0)	94.0 (84.8-97.8)	73.6 (57.8-85.0)	55.6 (37.1-72.7)
**>1000 % (95% CI)**	77.1 (56.7-89.7)	95.1 (87.7-98.1)	79.1 (65.4-88.4)	65.5 (47.8-79.7)

CI, confidence interval.

Supplementary Table 6 Sensitivity and specificity for detection of cancer in colorectal polyps, measured before (baseline) and after (post-intervention) the two-minute Blink-feature educational intervention, stratified by participant piecemeal endoscopic mucosal resection (pEMR) experience.

Polypectomy experience	Sensitivity baseline	Sensitivity post-intervention	Specificity baseline	Specificity post-intervention
**0**<br>**% (95% CI)**	59.6 (30.3-83.3)	88.0 (65.8-96.6)	69.4 (46.9-85.4)	54.8 (30.8-76.8)
**0-50**<br>**% (95% CI)**	76.1 (56.1-88.8)	95.4 (88.4-98.3)	66.8 (50.7-79.8)	46.1 (29.3-63.8)
**51-200**<br>**% (95% CI)**	72.7 (52.4-86.5)	91.8 (81.3-96.6)	75.7 (61.7-85.8)	62.8 (46.4-76.7)
**201-400**<br>**% (95% CI)**	70.9 (46.9-87.1)	95.7 (85.4-98.9)	79.1 (63.2-89.3)	61.1 (35.5-81.8)
**401-999**<br>**% (95% CI)**	69.5 (48.6-84.6)	93.3 (82.2-97.7)	83.3 (71.5-90.9)	66.1 (45.8-81.8)
**> 1000**<br>**% (95% CI)**	81.2 (55.7-93.7)	97.7 (90.2-99.5)	84.2 (68.1-93.0)	70.0 (43.5-87.5)

CI, confidence interval.

Supplementary Table 7 Sensitivity and specificity for detection of invasive cancer in colorectal polyps according to number of Blink features identified per lesion.

Number of blink features	Sensitivity	Specificity
0	100.0%	0.0%
(95% CI)	(100.0-100.0)	(0.0-0.0)
≥ 1	95.8%	30.4%
(95% CI)	(89.1- 98.8)	(20.2-41.5)
≥ 2	78.2%	70.5%
(95% CI)	(59.9-90.8)	(57.3-80.8)
≥ 3	47.6%	92.4%
(95% CI)	(27.2-69.2)	(85.5-96.3)
≥ 4	19.3%	98.8%
(95% CI)	(7.8-38.0)	(97.0-99.6)
≥ 5	4.6%	99.9%
(95% CI)	(1.3-12.8)	(99.7-100.0)
≥ 6	0.5%	100.0%
(95% CI)	(0.1-2.0-)	(100.0-100.0)

CI, confidence interval.

Supplementary Table 8 Performance of multivariate model shown in Table 2 assessed by 5-fold cross validation.

Fold	AUC	Accuracy	Sensitivity	Specificity	Threshold
1	0,799	73,0%	80,9%	68,6%	0,297
2	0,809	69,4%	90,0%	57,7%	0,279
3	0,774	68,5%	85,1%	59,7%	0,28
4	0,764	69,1%	77,5%	64,6%	0,304
5	0,829	71,5%	89,1%	62,6%	0,285
Average	0,795	70,3%	84,5%	62,6%	0,289

AUC, area under the curve.

Supplementary table 9 Agreement and reliability metrics for the six Blink features. ICC: intraclass correlation coefficients

	**Agreement**	**Fleiss kappa (with interpretation)**	**ICC overall**
Fold deformation	0.57	0.14 (slight)	0.45
Extra redness	0.54	0.34 (fair)	0.63
Depression	0.53	0.31 (fair)	0.54
Chicken skin	0.85	0.21 (fair)	0.46
Ulceration	0.88	0.21 (fair)	0.53
Spontaneous bleeding	0.94	0.51 (moderate)	0.75

Supplementary Fig. 1 Discriminative and calibration performance of the predictive model. Left panel shows the receiver operating characteristic (ROC) curve with an area under the curve (AUC) of 0.79. The right panel displays the calibration plot comparing predicted probabilities to observed proportions, with predictions binned into deciles. The dashed diagonal line indicates perfect calibration. Deviations from this line reflect over- or under-estimation of risk.

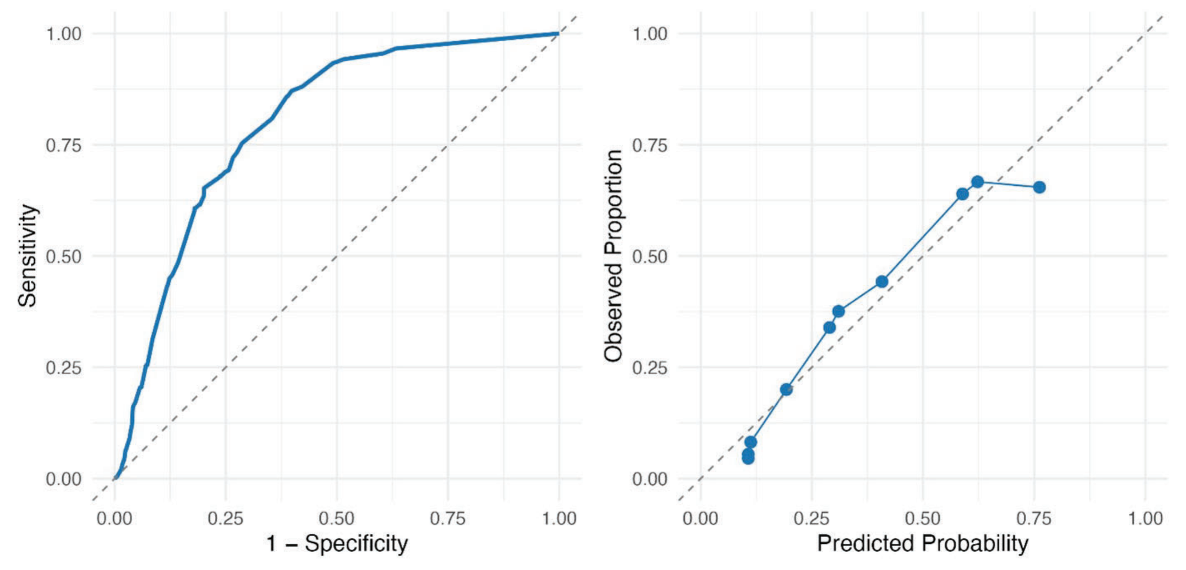

References

1 *Ikehara H, Saito Y, Matsuda T* et al. Diagnosis of depth of invasion for early colorectal cancer using magnifying colonoscopy. J Gastroenterol Hepatol 2010; 25: 905-912
2 *Uraoka T, Saito Y, Matsuda T* et al. Endoscopic indications for endoscopic mucosal resection of laterally spreading tumours in the colorectum. Gut 2006; 55: 1592-1597
3 *Jang HW, Park SJ, Cheon JH* et al. Does magnifying narrow-band imaging or magnifying chromoendoscopy help experienced endoscopists assess invasion depth of large sessile and flat polyps? Dig Dis Sci 2014; 59: 1520-1528
4 *Saitoh Y, Obara T, Watari J* et al. Invasion depth diagnosis of depressed type early colorectal cancers by combined use of videoendoscopy and chromoendoscopy. Gastrointest Endosc 1998; 48: 362-370
5 *Kudo S, Kashida H, Tamura T* et al. Colonoscopic diagnosis and management of nonpolypoid early colorectal cancer. World J Surg 2000; 24: 1081-1090
6 *Saito Y, Fujii T, Kondo H* et al. Endoscopic treatment for laterally spreading tumors in the colon. Endoscopy 2001; 33: 682-686
7 *Puig I, López-Cerón M, Arnau A* et al. Accuracy of the narrow-band imaging international colorectal endoscopic classification system in identification of deep invasion in colorectal polyps. Gastroenterology 2018
8 *Uno Y, Munakata A*. Endoscopic and histologic correlates of colorectal polyp bleeding. Gastrointest Endosc 1995; 41: 460-467
9 *Bugajski M, Kaminski MF, Orlowska J* et al. Suspicious macroscopic features of small malignant colorectal polyps. Scand J Gastroenterol 2015; 50: 1261-1267
10 *Matsuda T, Saito Y, Hotta K* et al. Prevalence and clinicopathological features of nonpolypoid colorectal neoplasms: should we pay more attention to identifying flat and depressed lesions? Dig Endosc 2010; 22: S57-S62
11 *Horie H, Togashi K, Kawamura YJ* et al. Colonoscopic stigmata of 1 mm or deeper submucosal invasion in colorectal cancer. Dis Colon Rectum 2008; 51: 1529-1534
12 *Misawa M, Kudo SE, Wada Y* et al. Magnifying narrow-band imaging of surface patterns for diagnosing colorectal cancer. Oncol Rep 2013; 30: 350-356
13 *Shatz BA, Weinstock LB, Thyssen EP* et al. Colonic chicken skin mucosa: an endoscopic and histological abnormality adjacent to colonic neoplasms. Am J Gastroenterol 1998; 93: 623-627
14 *Lee YM, Song KH, Koo HS* et al. Colonic chicken skin mucosa surrounding colon polyps is an endoscopic predictive marker for colonic neoplastic polyps. Gut Liver 2022; 16: 754-763
15 *Nowicki MJ, Bishop PR, Subramony C* et al. Colonic chicken-skin mucosa in children with polyps is not a preneoplastic lesion. J Pediatr Gastroenterol Nutr 2005; 41: 600-606
16 *Guan J, Zhao R, Zhang X* et al. Chicken skin mucosa surrounding adult colorectal adenomas is a risk factor for carcinogenesis. Am J Clin Oncol 2012; 35: 527-532
17 *Kudo S, Hirota S, Nakajima T* et al. Colorectal tumours and pit pattern. J Clin Path 1994; 47: 880-885

18 *Bates D, Mächler M, Bolker B* et al. Fitting linear mixed-effects models using lme4. J Stat Software 2015; 67: 1- 48
19 *Russell VL*, 2024. emmeans: Estimated Marginal Means, aka Least-Squares Means. https://CRAN.R-project.org/package=emmeans
